# The use of opioids at the end of life: the knowledge level of Dutch physicians as a potential barrier to effective pain management

**DOI:** 10.1186/1472-684X-9-23

**Published:** 2010-11-12

**Authors:** Mette L Rurup, Christiaan A Rhodius, Sander D Borgsteede, Manon SA Boddaert, Astrid GM Keijser, H Roeline W Pasman, Bregje D Onwuteaka-Philipsen

**Affiliations:** 1 VU University Medical Center, EMGO Institute for Health and Care Research, Department of Public and Occupational Health, Amsterdam, The Netherlands; 2 VU University Medical Center, Palliative Care Center of Expertise, Amsterdam, The Netherlands; 3 VU University Medical Center, Department of Medical Oncology, Palliative Care Consultation team, Amsterdam, The Netherlands; 4 VU University Medical Center, Department of Clinical Pharmacology and Pharmacy, Amsterdam, The Netherlands; 5 Department of Clinical Pharmacy, Academic Medical Center, Amsterdam, The Netherlands

## Abstract

**Background:**

Pain is still one of the most frequently occurring symptoms at the end of life, although it can be treated satisfactorily in most cases if the physician has adequate knowledge. In the Netherlands, almost 60% of the patients with non-acute illnesses die at home where end of life care is coordinated by the general practitioner (GP); about 30% die in hospitals (cared for by clinical specialists), and about 10% in nursing homes (cared for by elderly care physicians).

The research question of this study is: what is the level of knowledge of Dutch physicians concerning pain management and the use of opioids at the end of life?

**Methods:**

A written questionnaire was sent to a random sample of physicians of specialties most often involved in end of life care in the Netherlands. The questionnaire was completed by 406 physicians, response rate 41%.

**Results:**

Almost all physicians were aware of the most basal knowledge about opioids, e.g. that it is important for treatment purposes to distinguish nociceptive from neuropathic pain (97%). Approximately half of the physicians (46%) did not know that decreased renal function raises plasma concentration of morphine(-metabolites) and 34% of the clinical specialists erroneously thought opioids are the favoured drug for palliative sedation.

Although 91% knew that opioids titrated against pain do not shorten life, 10% sometimes or often gave higher dosages than needed with the explicit aim to hasten death. About half felt sometimes or often pressured by relatives to hasten death by increasing opioiddosage.

The large majority (83%) of physicians was interested in additional education about subjects related to the end of life, the most popular subject was opioid rotation (46%).

**Conclusions:**

Although the basic knowledge of physicians was adequate, there seemed to be a lack of knowledge in several areas, which can be a barrier for good pain management at the end of life. From this study four areas emerge, in which it seems likely that an improvement can improve the quality of pain management at the end of life for many patients in the Netherlands: 1)palliative sedation; 2)expected effect of opioids on survival; and 3) opioid rotation.

## Background

Many factors may hamper optimal pain management such as patient nonadherence to drug therapy, underreporting of pain or miscommunication between patient and caregivers; from a healthcare provider perspective, inadequate assessment of pain, poor documentation and miscommunication may limit optimal pain management [1]. To achieve good patient care at the end of life, also adequate knowledge of pain management and opioids among physicians is essential. Pain is still one of the most frequently occurring symptoms at the end of life, although it can be treated satisfactorily in most cases if the physician has adequate knowledge [2-7].

Among countries, different physicians are involved in pain treatment at the end of life. In the Netherlands, health care is characterised by its strong emphasis on primary care, where the family physician is the central professional in the management and coordination of the patient's care [8]. Almost 60% of the patients with non-acute illnesses die at home where palliative care is coordinated by the general practitioner (GP) [9]. About 30% of the patients with non-acute illnesses die in hospitals (cared for by clinical specialists), and about 10% in nursing homes (where the elderly care physician - formerly known as nursing home physician - is responsible) [9]. It is estimated that 5% of all people die in a specialised palliative care unit. These units are part of nursing homes or homes for the elderly (50%), or hospices (50%) [10]. Characteristic for the Netherlands is that euthanasia is a legal option when suffering is unbearable and hopeless; in 2005, 1.7% of all deaths was the result of euthanasia [11].

Elderly care physicians usually work in a single organisation, and are trained to care for elderly patients, including end of life care [12,13]. A wide range of clinical specialists provide end of life care in hospitals, and they also consult their colleagues in general practice and nursing homes to discuss complex symptoms in end of life care: for example 55% of the GPs cooperate with a clinical specialist [14]. In palliative home care, GPs frequently cooperate with district nurses[14] and can consult a regionally working palliative care consultation team [15].

In all these settings, possibilities to treat patients at the end of life with medication have increased in the past decade. Being able to take advantage of these possibilities does require physicians to maintain their knowledge. Guidelines that are developed by medical professional organizations can be a useful tool for this purpose.

Initially, there was optimism that the availability of evidence-based guidelines about pain management at the end of life would lead to great improvements in care. Research in the US showed that the problem of suboptimal pain management was far more complex than previously suspected: certain myths about the benefit of pain and the disadvantages and limitations of treatment of pain are to such an extent assimilated in the collective knowledge, that they are not banished by simply stating in a guideline that they are false [16]. Examples of such widespread myths are that pain often is inevitable, that administering increasing dosages of opioids hastens the end of life, and that adverse effects of opioids, such as drowsiness and confusion are inevitable [16-24].

In the international literature, the main focus of the effect of such myths is that opioids are underdosed, as a consequence of which patients may suffer needlessly [16,20,21]. Studies in the Netherlands have also shown that physicians treating cancer pain do not always follow evidence-based guidelines [25-27]. Another risk of myths can be that physicians overdose opioids, e.g. if they intend to hasten the end of life, and think they can attain this goal by increasing the dosage further than is required for pain management.

Developing and dispersing evidence-based guidelines is an important step towards an evidence-based practice, but further steps are required. To improve the quality of care at the end of life, it is subsequently necessary to study which knowledge has and which has not permeated to physicians, and which myths may hinder further permeation.

We aimed to study the level of knowledge of physicians, including both knowledge that should be familiar ground to all physicians, and facts that have more recently been added to the body of evidence, but should also be known to physicians involved in end of life care. Furthermore, we aimed to study experiences of physicians with pain management and opioids at the end of life, and possible barriers to good pain management at the end of life according to physicians.

## Methods

Medical ethical approval was not necessary for this study in the Netherlands because it was a cross-sectional questionnaire study among physicians, patients were not involved and no interventions were done.

### Developing the questionnaire and the pilot study

For this study, we developed a questionnaire to investigate knowledge and attitude concerning opioids and pain management at the end of life, based upon previous studies [19,20,28-34]. Questions were adapted and added to be able to answer the research questions, and to fit the specific situation in the Netherlands. For this purpose we used Dutch guidelines, original articles and review articles [17,22,23,35-53].

The questionnaire consisted of three types of questions: 1) knowledge statements with three answering options: "true", "false" and "don't know" 2) experience and attitude questions with five answering options: "completely agree", "agree more than disagree", "neutral", "disagree more than agree" and "completely disagree" and 3) other experience and attitude questions, partly structured with four answering options: "often", "sometimes", "seldom" and "never" and partly semi-structured. Furthermore several background characteristics were asked, including to grade their own knowledge about opioids and pain management by scoring 1-10. After completing the knowledge statements, respondents were asked to again grade their own knowledge.

The questionnaire was piloted among general practitioners in training (n = 54) and elderly care physicians in training (n = 24). Most physicians in the pilot indicated they used 15 minutes to complete the questionnaire. Opinions differed on the difficulty level of the knowledge statements in the questionnaire, but most physicians (79%) did not think the statements were too difficult or they even thought they were (too) easy. The translated questionnaire is presented as an appendix in additional file 1.

### Sampling frame

Physicians from the specialties which are most involved in pain management at the end of life were included in the random samples, namely: general practitioners, elderly care physicians, and clinical specialists specialized in internal medicine (incl oncologists, excl gastrointestinal and hepatic physicians), pulmonology, surgery (excl orthopaedics and plastic surgeons), cardiology, neurology, and anaesthesia. Geriatricians were not included in the sample as this is a small specialism in the Netherlands. The samples were randomly taken from different sources: general practitioners from the Netherlands Institute of Health Services Research (NIVEL), elderly care physicians from the Association of Physicians specialized in care for older people (Verenso) and clinical specialists from the medical address guide (BSL). These registrations consist of virtually all general practitioners, elderly care physicians and clinical specialists in the Netherlands. Including criteria were: currently working in the Netherlands as a physician, not in training.

### Data collection and anonymity

The questionnaire was sent in January 2009. Two reminders were sent. The first reminder was a letter or an e-mail to remind them of the study, the second reminder included the questionnaire again and a short non-response questionnaire. Respondents could complete the questionnaire on paper or online. The written questionnaire was anonymous. A separate answer card with the personal data of the respondent could be mailed separately by the respondent to prevent further reminders. The online questionnaire was not anonymous, because it would otherwise not be possible to exclude the possibility that people who were not in the sample would complete the questionnaire. When the database was closed the questionnaires were made anonymous.

After the closing of the data collection, a document with the correct answers to the statements to test knowledge was sent by e-mail to the respondents who had indicated they were interested to receive this.

### Analysis

All returned questionnaires were included in our analysis. Differences between non-responders and responders, and between general practitioners, elderly care physicians and clinical specialists were calculated using chi square tests. A significance level of p < 0.05 was used. Answers "completely agree" and "agree more than disagree" were merged to the category "agree" when describing attitudes and experiences concerning pain, the prescription of opioids and consultation. A linear regression analysis was done with as a dependent variable the number of correct answers to the knowledge statements. The analysis was done backward stepwise until all independent variables had a p < 0.05 to construct a predictive model.

## Results

### Background characteristics

In Table 1 the background characteristics of the respondents are shown per specialty. Clinical specialists are employed full-time more often than general practitioners and elderly care physicians, and clinical specialists had most experience with prescribing opioids. Elderly care physicians had more experience with non-sudden deaths than clinical specialists and general practitioners and elderly care physicians had most often received a specific education in palliative care, apart from the regular medical education. Patients who were treated by the general practitioner at the moment of death were prescribed opioids more often than patients treated by clinical specialists or elderly care physicians at the moment of death.

**Table 1:** Background characteristics of respondents

	**General practitioners**	**Elderly care physicians**	**Clinical specialists**	p-value*	**Total**
	**n = 182**	**n = 110**	**n = 112**		**n = 406^†^**
	**%**	**%**	**%**		**%**
	
**Gender**				<0.05	
• Men	63	36	70		58
• Women	36	65	30		42
**Age (years)**				≥0.05	
• <40	20	31	13		21
• 41-50	32	33	40		35
• 51-60	41	35	41		39
• >60	7	1	6		5
**Employment**				<0.05	
• full-time	54	27	80		55
• part-time	43	71	20		45
**Grade given for own knowledge *before *completing the questionnaire (1-10)**				<0.05	
• <5.5	3	2	9		4
• 5.5-6.5	13	6	15		11
• 6.6-7.5	56	52	39		50
• 7.6-8.5	27	38	29		30
• 8.6-10	2	3	8		4
**Grade given for own knowledge *after *completing the questionnaire (1-10)**				<0.05	
• <5.5	2	1	13		5
• 5.5-6.5	10	9	17		12
• 6.6-7.5	53	53	39		49
• 7.6-8.5	33	34	28		32
• 8.6-10	2	3	5		3
**Received specific education in palliative care apart from regular medical education**	77	90	43	<0.05	71
**Number of patients to whom the respondent had prescribed opioids in 2008**				≥0.05	
• None	0	2	3		1
• 1-5	5	2	13		6
• 6-20	43	28	24		33
• 21-50	35	46	20		34
• >50	16	21	42		25
**Number of deaths after a sickbed (non-sudden) in 2008 while being treating physician**				<0.05	
• None	0	0	11		3
• 1-5	35	5	15		21
• 6-20	59	39	49		50
• 21-50	6	47	17		21
• >50	1	9	9		5
**Percentage of these patients that were using opioids at the moment of death^‡^**				<0.05	
• None	1	1	8		2
• 1-40%	17	16	36		22
• 41-80%	46	66	42		51
• 81-100%	37	17	14		25

* chi-square test testing differences between the three groups of physicians

**† **including 2 physicians who did not specify their specialty

**‡ **percentage of patients *per physician*, (e.g. 37% of the general practitioners indicated that 81-100% of their patients were using opioids at the moment of death), percentages of physicians who had at least one death after a sickbed (non-sudden) in 2008

### Response rate and non-response analysis

In total 1044 physicians were sent a questionnaire, 55 envelopes were returned to sender, leaving 989 possible respondents. Of the 400 general practitioners, 182 completed the questionnaire (response rate 46%), of the 175 elderly care physicians, 110 completed the questionnaire (response rate 63%), and of the 414 clinical specialists, 112 completed the questionnaire (response rate 27%), 2 physicians completed the questionnaire, without specifying their specialty. The total response rate was 406/989 = 41%. Of the completed questionnaires, 80% was completed in writing and 20% was completed online. The non-response questionnaire was completed by 29 general practitioners, 6 elderly care physicians, and 43 clinical specialists.

There were several significant differences between physicians who completed the non-response questionnaire (n = 78) and physicians who completed the full questionnaire (n = 406) (not in Table). The non-response questionnaire was completed significantly more often by clinical specialists (55% of the non-response questionnaires were completed by clinical specialists vs. 28% of the full questionnaires). Physicians who completed the non-response questionnaire were significantly more often employed full-time than physicians who completed the full questionnaire (80% vs. 55%), but had significantly less experience with prescribing opioids (85% vs. 99% in the past year) and with care at the end of life (84% vs. 97% in the past year). Furthermore, the full questionnaire was significantly more often completed by women than the non-response questionnaire. Finally, physicians who completed the non-response questionnaire rated their own knowledge of opioids and pain management on average somewhat lower than physicians who completed the full questionnaire (6.8 vs. 7.2), but this difference was not statistically significant.

The most important reasons for non-response were "too busy", "no time" and "too many studies". Other reasons for non-response were lack of experience with care at the end of life in 18% and 12% thought they lacked the knowledge to complete the questionnaire.

### Knowledge statements

In Table 2 the answers to the knowledge statements are shown per specialty. Of the 14 presented statements, general practitioners and elderly care physicians gave on average between 10 and 11 correct answers, clinical specialists gave on average between 9 and 10 correct answers. With statements 3, 6, 7, 9 and 10 more than 20% of the physicians marked that the statement was true when it was false, or the other way around. The answers of clinical specialists were significantly less often correct than the answers of general practitioners and/or elderly care physicians on the statements 1,2,9 and 11-14.

**Table 2:** Answers to the knowledge statements per specialty*

		General practitioners	Elderly care physicians	Clinical specialists	p-value^†^	Total
		n = 182	n = 110	n = 112		n = 406
Average number of correct answers		10,4	10,6	9,5		10,2
		%	%	%		%
**Pain**						

1. In the management of pain it is important to differentiate between nociceptive and neuropathic pain	**% true** % false % don't know	**98** 1 1	**100** 0 0	**94** 0 6	<0.05	**97** 1 2
2. Administration of opioids early on in the disease hampers good pain control later on in the disease process	% true **% false** % don't know	3 **96** 2	8 **91** 1	10 **84** 6	<0.05	6 **91** 3
3. Opioids may cause or worsen pain	**% true** % false % don't know	**26** 45 29	**34** 43 23	**35** 43 22	≥0.05	**30** 45 25

**Prescribing opioids**						

4. Once opioids have been started, other analgesics should be discontinued	% true **% false** % don't know	4 **96** 1	1 **99** 0	5 **92** 3	≥0.05	4 **96** 1
5. Opioids are only indicated for cancer patients	% true **% false** % don't know	0 **100** 0	0 **100** 0	2 **98** 0	≥0.05	0 **100** 0
6. Simultaneous prescription of a weak opioid (e.g. tramadol) and a strong opioid (e.g. morphine) is contra-indicated^**‡**^	**% true** % false % don't know	**49** 28 22	**57** 26 17	**57** 29 14	≥0.05	**54** 28 19
7. Decreased renal function raises plasma concentration of morphine(-metabolites)	**% true** % false % don't know	**51** 22 27	**55** 23 22	**57** 23 19	≥0.05	**54** 22 23
8. Opioids have a maximum dosage	% true **% false** % don't know	4 **94** 2	4 **93** 3	8 **88** 4	≥0.05	5 **92** 3

**Side-effects**						

9. Life-threatening respiratory depression is a real danger when titrating morphine against pain	% true **% false** % don't know	16 **83** 2	19 **75** 6	31 **68** 1	<0.05	21 **77** 3
10. Drug management of nausea in treatment with opioids is evidence-based	% true **% false** % don't know	40 **18** 42	50 **12** 38	34 **17** 50	≥0.05	41 **16** 43

Opioid rotation*						

11. You want to change a daily dosage of 60 mg oxycodon to a fentanyl patch with an equivalent dosage. The strength of the patch is.^**^	**% 25 μg p/h^†^** **% 50 μg p/h^†^** % false % don't know	**28** **38** 6 28	**37** **34** 12 17	**25** **24** 11 41	<0.05	**30** **33** 9 28

**Sedation and shortening of life by opioids**						

12. Opioids titrated against pain, shorten life	% true **% false** % don't know	3 **96** 1	2 **95** 3	14 **81** 6	<0.05	6 **91** 3
13. Opioids are the favoured drugs for palliative sedation	% true **% false** % don't know	13 **86** 1	5 **95** 0	34 **57** 8	<0.05	17 **81** 3
14. Opioids are appropriate drugs to perform euthanasia	% true **% false** % don't know	0 **99** 1	0 **97** 3	8 **86** 6	<0.05	2 **95** 3

* correct answer is printed in bold

^**†**^chi-square test testing differences between the three groups of physicians

^**‡ **^a simultaneous prescription of a weak and a strong opioid is not a contra-indication in the true sense of the word. It is, however, for pharmacodynamic reasons in general not a sensible combination. This is why it is not advocated in the available guidelines for treatment of pain.

** physicians could circle the following options 12/25/50/75/100/125/150 μg per hour or "don't know". With this question 2 answers were considered correct, because different guidelines give different conversions, which leads to two different answers

### Attitudes concerning pain, the prescription of opioids and asking for advice

The majority of the physicians (60%) found good pain control in practice complex (Table 3). If physicians needed advice about pain management, they consulted their direct colleagues (74%), their pharmacist (53%) or the palliative care consultation team (not in Table). Most physicians did know of the availability of the consultation team (90%), but 40% had never used it. The large majority (95%) indicated they were open to unsolicited advice from the pharmacist.

**Table 3:** Attitudes and experiences concerning pain, the prescription of opioids and consultation

	General practitioners	Elderly care physicians	Clinical specialists	p-value*	Total
	n = 182	n = 110	n = 112		n = 406^†^
	% agree	% agree	% agree		% agree
**Pain**					

• In case of a change in pain symptomatology, I always take a comprehensive pain history	74	64	73	≥0.05	70
• In practice I find good pain control complex	60	56	65	≥0.05	60
• With the current medical possibilities, pain is always controllable	21	26	29	≥0.05	24
• When a patient is in pain, he/she will always indicate this	16	8	17	≥0.05	14

**Prescribing opioids**					

• When prescribing opioids, I always prescribe a maintenance dosage plus a dosage to be used when needed (break-through medication)	90	68	84	≥0.05	80
• Nursing/care staff are reluctant to administer the opioids I prescribe	4	4	10	≥0.05	6
• I try to delay the prescription of opioids for as long as possible	4	9	7	≥0.05	6

**Consultation**					

• Inadequate support from the pharmacist, hampers pain management	7	8	3	≥0.05	6
• Asking for consultation feels like personal defeat	2	2	4	≥0.05	2
	**% yes**	**% yes**	**% yes**		**% yes**

**Laxative and anti-emetic**					

• As a general rule, I combine the prescription of an opioid with a laxative	94	69	76	<0.05	83
• As a general rule, I combine the prescription of an opioid with an anti-emetic	8	2	13	≥0.05	8

* chi-square test testing differences between the three groups of physicians

^**† **^including 2 physicians who did not specify their specialty

The majority of the physicians (83%) indicated that they combined opioids with a laxative. Clinical specialists did not see constipation often as a side-effect. Elderly care physicians indicated that it was not always possible to prescribe opioids to their patients because it was not necessary or because it would be too burdening.

The majority of the physicians (92%) indicated that they did not combine opioids with an anti-emetic. Many physicians said they waited to see whether the patient developed nausea.

### Attitudes and experiences concerning opioid rotation, tolerance, addiction and shortening of life by opioids

Table 4 shows that in practice, clinical specialists less often rotate opioids than general practitioners and elderly care physicians (47% versus 70% and 66% often or sometimes). A majority of all physicians (74%), especially among clinical specialists (85%) had sometimes or frequently experienced that tolerance could occur with the use of opioids, but less frequently thought tolerance hampered the usage of opioids in pain control (20%).

**Table 4:** Attitudes and experiences concerning opioid rotation*, tolerance**, addiction and shortening of life by opioids

	General practitioners	Elderly care physicians	Clinical specialists	**p-value**^†^	Total n = 406
	n = 182	n = 110	n = 112		n = 406^†^
	%often/sometimes	%often/sometimes	%often/sometimes		%often/sometimes
Opioid rotation*					

• I rotate opioids in practice	70	66	47	<0.05	62
• I rotate opioids if pain control is inadequate	77	72	65	≥0.05	72
• I rotate opioids in case of side-effects	67	73	63	≥0.05	67
• I find calculating of opioid dosages when rotating difficult	62	57	57	≥0.05	59

Tolerance** and fear of addiction					

• I have noticed that tolerance can develop in the usage of opioids	68	74	85	<0.05	74
• Tolerance hampers the usage of opioids in pain control	15	20	29	<0.05	20
• Patients' fear of addiction hampers the usage of opioids in practice	49	35	51	<0.05	46

**Shortening of life by opioids**					

• It occurs that relatives of a patient or other persons concerned, put pressure on me to increase the opioids in the hope of hastening death	36	75	50	<0.05	50
• When titrating the dosage of opioids upwards against pain, I take into account that this may hasten the death of the patient	38	44	68	<0.05	48
• It occurs that I increase the dosage of opioids to a level above that of what is needed for pain and symptom control with the explicit aim to hasten the death of the patient	11	1	19	<0.05	10

* The following definition of tolerance was given in the questionnaire: "By tolerance for a drug we mean that a patient needs a higher dose to reach the same pain relief while the pain stimulus remains the same. Tolerance has proven to be difficult to measure in practice, we are interested in your personal experience."

^† ^chi-square test testing differences between the three groups of physicians

^**‡ **^including 2 physicians who did not specify their specialty

** The following definition of opioid rotation was given in the questionnaire: "With the term "opioid rotation" we mean the replacing of one opioid by another opioid."

Elderly care physicians experienced pressure from people around the patient to increase the opioids in the hope of hastening death significantly more often than general practitioners and clinical specialists (75% vs. respectively 36% and 50% sometimes or often). One in 10 physicians at least sometimes increased the dosage of opioids above the level needed for pain and symptom control with the explicit aim to hasten the death of the patient. Clinical specialists and general practitioners did this more often than elderly care physicians (respectively 19% and 11% vs. 1%, Table 4).

### Side-effects

Most commonly observed side effect were constipation (observed 86%), nausea (33%), drowsiness (30%), and delirium (9%). Although less than 1% of the physicians reported often observing life-threatening respiratory depression, it was observed seldom by 61% of the clinical specialists. General practitioners (60%) and elderly care physicians (62%) said they never observed this possible side-effect.

### Education and self-knowledge vs. knowledge

In Table 5 the relation is shown between education and the estimates of own knowledge vs. the number of correct answers to the 14 knowledge statements. Physicians who answered more knowledge statements correctly, had more often received specific training in palliative care.

**Table 5:** Relation between education and the estimates of own knowledge vs. the number of correct answers to the 14 knowledge statements

	**3-9 correct answers**	**10 correct answers**	**11 correct answers**	**12-14 correct answers**	p-value*
	**N = 114**	**N = 95**	**N = 105**	**N = 93**	
	**%**	**%**	**%**	**%**	
	
Received specific education in palliative care	60	68	78	87	<0.05
**Grade given for own knowledge *before *completing the questionnaire (1-10)^† ^**					<0.05
• <5.5	11	1	1	3	
• 5.5-6.5	19	11	10	5	
• 6.6-7.5	46	60	51	46	
• 7.6-8.5	20	27	34	41	
• 8.6-10	5	1	5	4	
**Grade given for own knowledge *after *completing the questionnaire (1-10)^† ^**				≥0.05	<0.05
• <5.5	11	1	3	2	
• 5.5-6.5	24	6	8	6	
• 6.6-7.5	48	61	50	41	
• 7.6-8.5	15	32	37	46	
• 8.6-10	2	0	4	6	
Are there enough options for you for additional education about opioids and pain management?					<0.05
• Yes	77	94	93	95	
• No	23	6	7	5	

* chi square test to test differences in physician characteristics for physicians with different numbers of correct answers (categorized in 4 groups)

^† ^Physicians graded their own knowledge about opioids and pain management by scoring 1-10. After completing the knowledge statements, respondents were asked to again grade their own knowledge. Physicians could not see how they did on the knowledge statements, they did not receive the answers to the knowledge statements immediately after completing the questionnaire.

Physicians who graded their own knowledge about pain management and opioids higher, answered more knowledge statements correctly. About a quarter of the physicians who answered one to nine of the knowledge statements correctly lowered their grade when asked again at the end of the questionnaire.

The majority of the physicians (83%) would like to receive additional education about one or more subjects related to the end of life (not in Table). Almost half of the physicians (46%) indicated they were interested in opioid rotation, pain- and symptom management in general (44%), palliative sedation (30%), pharmacological mechanism of opioids (18%), side-effects (16%), and other subjects (5%). Some physicians who said there were not enough options for education said that offerings for education often came from the pharmaceutical industry, that education did not include the subjects they were interested in, that there was not a lot of education in the form of courses or that courses were not in their region. For the majority, the preferred way of education was symposia or conferences (60%) and literature in Dutch journals (52%).

### Linear regression analysis

A linear regression analysis was done, with as the dependent variable the number of correct answers to the knowledge statements. Background characteristics and experience were included as independent variables: specialty of the physician (general practitioner/elderly care physician/clinical specialist), gender, year of graduation, full-time or part-time employment, specific education in palliative care, number of patients to whom opioids had been prescribed in the past year, number of patients who had died non-suddenly in the past year. The resulting model (R^2 ^= 0.16) showed that more experience with prescribing opioids and having received a specific education in palliative care have a positive influence on the number of correctly answered knowledge statements. Furthermore the variable "clinical specialist"(vs. general practitioner and elderly care physician) had an independent negative influence on the number of correctly answered statements. Finally, men answered less statements correctly than women did. (Table 6).

**Table 6:** Physician characteristics related to the number of correct answers on the knowledge statements (multivariate linear regression; n = 406)

Dependent variable	Independent variables*	Beta (standardised regression coefficient)	p
• Number of correct answers on the knowledge statements	• Number of patients to whom the respondent prescribes opioids per year	.22	<0.001
	• Received specific education in palliative care	.18	<0.001
	• Clinical Specialist	-.16	<0.001
	• Man	-.10	0.03

* variables that were analysed, but were not significant in the final model: year of graduation, full-time or part-time employment, number of patients that had died non-suddenly in the past year

## Discussion

Studying the knowledge level of physicians concerning pain management presents a number of challenges, because pain management is not a "hard science". It cannot be mastered by knowing a number of facts, but this method of study only allows for testing such facts. We have limited the knowledge statements almost exclusively to knowledge about which consensus has been reached and which is incorporated in pain management guidelines. Even then, it is difficult to make true or false statements. Physicians' whose answer is considered 'wrong' may have good reasons to think their answer is correct. We cannot set a limit of the number of statements that should be answered correctly to be an adequate physician.

We can conclude that several statements were answered correctly by the majority of the physicians. In our study physicians answered on average 10 out of 14 knowledge statements correctly (71%). In various studies physicians scored between 31% and 68% correct answers [29,30,54], although the overall scores in different studies cannot be compared as such because other statements were used.

We also identified some points where there was lack of knowledge in a considerable part of the physicians. E.g. less than one in three physicians was aware that opioids may cause or worsen pain. Although this is a rare effect the clinical effects can be significant if the dose of opioids is increased instead of decreased when a patient suffers from opioid-induced pain sensitivity [39-42]. There also seemed to be a lack of awareness that decreased renal function raises plasma concentration of morphine(-metabolites). If a patient has decreased renal function, certain opioids are preferred above others, to prevent side-effects [55,56]. It is also crucial that physicians involved in end of life care are aware that opioids are not the drug of choice for palliative sedation. More than one in three clinical specialists thought that opioids are the favoured drugs for palliative sedation. According to Dutch guidelines for palliative sedation, sedation with opioids should be considered malpractice [53].

### Hastening the end of life

Although the majority of the general practitioners and the elderly care physicians said they had never experienced life-threatening respiratory depression as a side-effect of opioids, part of the general practitioners and elderly care physicians (respectively 16% and 19%) did think this was a real danger, even when titrated against pain. Among clinical specialists even a third thought this was a real danger, although many studies have shown that this is not the case [22,23,49-53]. However, this knowledge seems to conflict with practice as 48% of all physicians take hastening of the patient's death into account when titrating opioids. And it appears that physicians act upon this misconception: 19% of the clinical specialists and 11% of the general practitioners indicated that they increased the dosage of opioids to a level above what is needed for pain and symptom control with the explicit aim to hasten the death of the patient.

A surprisingly large percentage of the elderly care physicians (75%) did feel pressured sometimes or often by relatives of a patient or other persons to increase the dosage of opioids in the hope of hastening death. Possibly this is related to the fact that elderly care physicians often care for patients with dementia who are unable to discuss medical decisions with their physician.

This result illustrates that good care at the end of life is more comprehensive than prescribing the right (dosage of) drugs. Contact and communication with relatives are crucial in palliative care. It also shows that the misconception that high dosages of opioids hasten the end of life probably also exists among relatives and patients. In practice, only an overdose of opioids could hasten the end of life. Even then hastening death is not likely, but there is an increased risk of serious side-effects, such as a delirium [22,23,49-53]. For that reason the Dutch guideline about euthanasia drugs states that not opioids but barbiturates followed by neuromuscular relaxants are to be used for euthanasia [57]. Nevertheless, this study shows that 8% of the clinical specialists does think opioids are appropriate drugs for euthanasia.

However, it seems that the proportion of physicians acting upon this misconception is decreasing the last years. The proportion of opioids as drugs in euthanasia deaths has decreased from 21.6% in 2001 to 16.2% in 2005[11], making it likely that the knowledge of opioids in end of life has increased.

### Opioid rotation

Rotation of opioids means that one opioid is substituted by another. This can be done in case of inadequate pain management or in case of side-effects. A quarter of the clinical specialists said they never rotated opioids. Many physicians said they had difficulties calculating opioid dosages when rotating (59%) and many physicians were interested in education about opioid rotation (46%). The evidence for equianalgesic conversion rates is limited, and equianalgesic doses vary among individuals under varying conditions [58]. Clinical characteristics such as organ dysfunction and age influence pharmacokinetics and pharmacodynamics. Scientific insights are developing and sometimes conflicting - for example concerning the influence of age on pharmacokinetics of transdermal fentanyl [59,60]. This might explain why standards mention different equianalgesic conversions for opioids. Two current Dutch guidelines present different conversion factors for some types of opioids. The cause of this difference is unclear; possibly there is no evidence to formulate unambiguous conversion factors. As a result of this, the knowledge statement about opioid rotation in this study has two possible answers. In our analyses, both answers were considered correct. In practice the reason for rotation is also relevant for the conversion factor: in case of rotation because of side-effects, it is recommended to convert to a lower than equivalent dose, in case of rotation because of inadequate pain relief it is recommended to rotate to an equivalent dose.

### Asking for advice and education

Physicians had on average reasonable knowledge about their own knowledge level. However, part of the physicians who did not answer many knowledge statements correct, did think their knowledge was adequate. It is important that physicians know their limits, so they know when to ask for assistance.

A remarkable high number of physicians (83%) was interested in additional education about opioids or pain management. It seems there are enough options for additional education in the Netherlands, but that this does not always fit the wishes of the physicians. Physicians were interested in efficient, objective education, preferably by parties other than the pharmaceutical industry.

### Strengths and limitations of this study

A strength of this study is that it is the first study in the Netherlands which has mapped the knowledge level of physicians about the use of opioids and pain management at the end of life. It has highlighted some important gaps in the knowledge of Dutch physicians. The results can be useful to improve (additional) education, which is necessary to improve the quality of care at the end of life. The statements used in this study can also be used for training purposes, and in studies in other countries with the same objectives as this study.

A limitation of this study is that only a limited number of themes are highlighted in which physicians should have adequate knowledge, and within those themes, not all aspects can be studied. Other limitations are that the response rate is moderate, and that the questionnaire has not been validated, although the pilot study did not reveal questions or answer categories that were not understood by the respondents. Hence, the answer category 'don't know' should be interpreted as physicians that did not know the answer to the question. Physicians who participated in this study, assessed their own knowledge somewhat higher than physicians who answered the non-response questionnaire, so probably this study tends to give a too positive image of the knowledge of physicians rather than a too negative image. It is possible that the knowledge about opioids and attitudes differ also within the three groups of physicians, as a result of different clinical specialty or experience in palliative care. We did not analyse possible differences, as we did not design our study to investigate these.

## Conclusions

Although the effects of a gap in the knowledge about opioids on the clinical practice differ per subject, we can conclude that most of the knowledge questioned in our study should be part of the basal knowledge physicians should have, and that a gap in this knowledge can be a barrier for adequate care at the end of life. Good care at the end of life is more comprehensive than adequate knowledge about pain management, but this is an essential part of it.

From this study -including physicians' attitudes and opinions as well as a test of their knowledge- three areas emerge, in which it seems likely that an improvement in these areas can improve the quality of pain management at the end of life for many patients in the Netherlands, namely: 1) palliative sedation, opioids are too often erroneously considered the appropriate drug for palliative sedation; 2) hastening the end of life with opioids, opioids are too often erroneously thought to shorten life, even when titrated against pain; 3) opioid rotation, opioid rotation is considered a difficult area by the majority of physicians, in which they would like additional education.

## Competing interests

The authors declare that they have no competing interests.

## Authors' contributions

MLR was involved in conception and design of the study, acquisition of data, analysis and interpretation of data, drafting of the manuscript and revision. CAR was involved in design of the study, acquisition of data, interpretation of data, revising of the manuscript. SDB was involved in conception and design of the study, analysis and interpretation of data, revising of the manuscript, drafting the revision. MSAB was involved in conception and design of the study, interpretation of data, revising of the manuscript. AGMK was involved in acquisition of data, analysis of data, revising of the manuscript. HRWP was involved in conception and design of the study, interpretation of data, revising of the manuscript. BDOP was involved in conception and design of the study, interpretation of data, revising of the manuscript, drafting the revision. All authors read and approved the final manuscript.

## Authors' information

MLR works as a senior researcher in the subject of end of life studies at the VU University Medical Center, EMGO Institute for Health and Care Research, Department of Public and Occupational Health.

CAR works as an elderly care physician, and at the time of the study, worked at the VU University Medical Center, Department of Medical Oncology, Palliative Care Consultation team.

MSAB works as a hospice physician, and at the time of the study, worked at the VU University Medical Center, Department of Medical Oncology, Palliative Care Consultation team.

AGMK works as a junior researcher at the VU University Medical Center, EMGO Institute for Health and Care Research, Department of Public and Occupational Health.

SDB works as a pharmacist at the Department of Clinical Pharmacy of the Academic Medical Center and at the time of the study as a researcher at the Department of Clinical Pharmacology and Pharmacy of the VU University Medical Center

HRWP works as a senior researcher in the subject of end of life studies at the VU University Medical Center, EMGO Institute for Health and Care Research, Department of Public and Occupational Health.

BDOP works as a professor in end-of-life research at the VU University Medical Center, EMGO Institute for Health and Care Research, Department of Public and Occupational Health.

## Pre-publication history

The pre-publication history for this paper can be accessed here:

http://www.biomedcentral.com/1472-684X/9/23/prepub

## Supplementary Material

Additional file 1 **Appendix 1 questionnaire**. An English translation of the study questionnaire Click here for file
