# Supplementary material for: The use of opioids at the end of life: the knowledge level of Dutch physicians as a potential barrier to effective pain management
Source: BMC Palliat Care. 2010 Nov 12;9:23. doi: 10.1186/1472-684X-9-23 (PMC3000381; doi:10.1186/1472-684X-9-23)
Supplement: Additional file 1 — Appendix 1 questionnaire. An English translation of the study questionnaire [file 1472-684X-9-23-S1.DOC]

# *Appendix 1: questionnaire*

**Opioids and pain management**

**at the end of life**


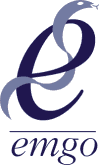

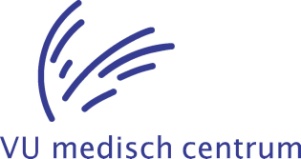

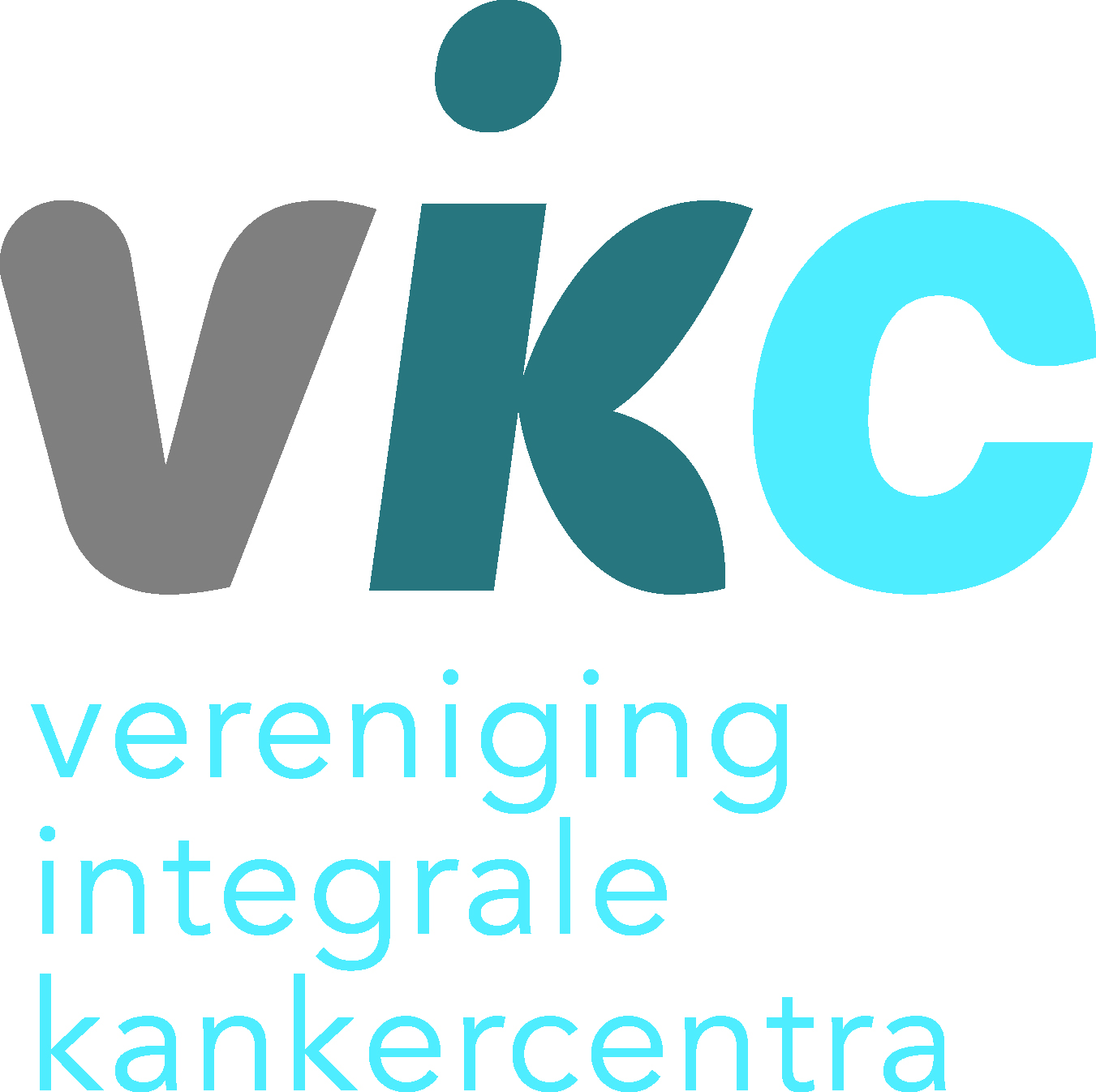


I. Characteristics

**1**. **Gender** ❑ female

❑ male

**2. Age** …………………… years

**3. University of medical education** ………………………………….

**4. Year of MD graduation** ………………..

**5. Specialisation** ❑ General practitioner

❑ Nursing home physician

❑ Medical specialist, specialisation:

❑ Surgery

❑ Internal medicine

❑ Pulmonology

❑ Cardiology

❑ Neurology

❑ Anaesthesiology

❑ Other: ……………….…

**6.** **Appointment** fulltime/ part time, for ……………... fte/ hours a week

**7. How many patients do you prescribe opioids yearly?**

none / 1-5 / 6-10 / 11-20 / 21-50 / > 50 *(number of patients/year*)

II. Pain

**1. How do you grade your own knowledge about opioids and pain management?**

……………………………*. (1 is lowest, 10 is highest )*

**2. Please answer the next statements with ‘true’, ‘false’, or ‘don’t know’.**

*Circle the correct answer.*

1. In the management of pain it is important to differentiate between nociceptive and neuropathic pain

true/ false/ don’t know

1. Administration of opioids early on in the disease hampers good pain control later on in the disease process

true/ false/ don’t know

1. Opioids may cause or worsen pain

true/ false/ don’t know

**3. Please indicate to what extend you agree with the next statements.**

Could you please answer these questions with your last terminal patient in mind?

|  | fully agree | agree | neutral | disagree | fully disagree |
| --- | --- | --- | --- | --- | --- |
| In case of a change in pain symptomatology, I always take a comprehensive pain history | ❑ | ❑ | ❑ | ❑ | ❑ |
| In practice I find good pain control complex | ❑ | ❑ | ❑ | ❑ | ❑ |
| With current medical possibilities, pain is always controllable | ❑ | ❑ | ❑ | ❑ | ❑ |
| When a patient is in pain, he/she will always indicate this | ❑ | ❑ | ❑ | ❑ | ❑ |

**4.** **Which of the next factors do you experience as limitating for good pain management?**

*(multiple answers possible)*

❑ Insufficiently detailed information about the pain

❑ Underreporting of pain by nursing staff

❑ Underreporting of pain by the patient

❑ Problems to gain insight in the experience of pain by the patient

❑ Nonadherence

❑ Opioid fear of the family

❑ Opioid fear of the patient

❑ My own reluctance to prescribe opioids

❑ Insufficient knowledge of good pain management

❑ Reluctance by nursing staff to give opioids

❑ Logistic problems (e.g. limited availability of home care / infusion pump)

❑ Other factors: ………………………………………………….

III. Opioids

**1. Please answer the next statements with ‘true’, ‘false’, or ‘don’t know’.**

**a.** Once opioids have been started, other analgesics should be discontinued

true/ false/ don’t know

**b.** Opioids are only indicated for cancer patients

true/ false/ don’t know

**c.** Simultaneous prescription of a weak opioid (e.g. tramadol) and a strong opioid (e.g. morphine) is contra-indicated

true/ false/ don’t know

**d.** Decreased renal function raises plasma concentration of morphine(-metabolites)

true/ false/ don’t know

**e.** Opioids have a maximum dosage

true/ false/ don’t know

**2.** **Please indicate to what extend you agree with the next statements.**

Please answer these questions with your last terminal patient in mind.

|  | fully agree | agree | neutral | disagree | fully disagree |
| --- | --- | --- | --- | --- | --- |
| When prescribing opioids, I always prescribe a maintenance dosage plus break-through medication when needed. | ❑ | ❑ | ❑ | ❑ | ❑ |
| Nursing/care staff are reluctant to administer the opioids I prescribe. | ❑ | ❑ | ❑ | ❑ | ❑ |
| I try to delay the prescription of opioids for as long as possible | ❑ | ❑ | ❑ | ❑ | ❑ |

1. **Could you give the most important reasons to postpone the initiation of an opioid prescription?**

*(multiple answers possible)*

❑ Due to the addictive effect of opioids

❑ Unsatisfactory results with prescribed opioids in previous patients

❑ To avoid shortening of life

❑ To avoid side effects

❑ Possibility that communication is hampered by drowsiness

❑ Reduced effectivess of opioids on pain with progress of underlying disease

❑ Preference of patient/family

❑ Follow the WHO pain ladder

❑ Other factor(s): ………………………………………………….

IV. Side effects

**1. Please answer the next statements with ‘true’, ‘false’, or ‘don’t know’.**

*Circle the correct answer.*

**a.** Life-threatening respiratory depression is a real danger when titrating morphine against pain.

true/ false/ don’t know

**b.** Drug management of nausea in treatment with opioids is evidence-based

true/ false/ don’t know

**2.** **How often do you register the next side effects of opioids in your practice?**

|  | often | sometimes | seldom | never |
| --- | --- | --- | --- | --- |
| Constipation | ❑ | ❑ | ❑ | ❑ |
| Nausea | ❑ | ❑ | ❑ | ❑ |
| Drowsiness | ❑ | ❑ | ❑ | ❑ |
| Delirium | ❑ | ❑ | ❑ | ❑ |
| Pruritus | ❑ | ❑ | ❑ | ❑ |
| Loss of cognition | ❑ | ❑ | ❑ | ❑ |
| Myoclonia | ❑ | ❑ | ❑ | ❑ |
| Bladder retention | ❑ | ❑ | ❑ | ❑ |
| Hyperaesthesia | ❑ | ❑ | ❑ | ❑ |
| Life threatening respiratory depression | ❑ | ❑ | ❑ | ❑ |
| Other side effects: …………………………… | ❑ | ❑ | ❑ | ❑ |

*Could you please answer the next questions with your last terminal patient in mind?*

**3. I combine the prescription of an opioid with a laxative.**

❑ Yes

❑ No, that is not my standard practice: *(multiple answers possible)*

❑ because I don’t encounter constipation that frequently as side effect that I think a fixed combination with a laxative is necessary

❑ because constipation is not always a problem when opioids are initiated

❑ but I do instruct the patient to mention changes in the defaecation pattern

❑ other, namely: ……………….……………………………………….

**4.** **I combine the prescription of an opioid with an anti-emetic drug.**

❑ Yes

❑ No, that is not my standard practice: *(multiple answers possible)*

❑ because I don’t encounter nausea that frequently as side effect that I think a fixed combination with an anti emetic drug is necessary

❑ but I do tell patients nausea is a side effect and that medication can be given

❑ other, namely: ……………….……………………………………….

V. Opioïd rotation

*With the term "opioid rotation" we mean the replacing of one opioid by another opioid.*

1. Please indicate how often (often, sometimes, seldom or never) the next statement are relevant in your practice

|  | often | sometimes | seldom | never |
| --- | --- | --- | --- | --- |
| I rotate opioids in general practics | ❑ | ❑ | ❑ | ❑ |
| I rotate opioids if pain control is inadequate | ❑ | ❑ | ❑ | ❑ |
| I rotate opioids in case of side-effects | ❑ | ❑ | ❑ | ❑ |
| I find calculating opioids dosages difficult when rotating | ❑ | ❑ | ❑ | ❑ |

1. **You would like to switch from oral oxycodon (60mg daily) to a transdermal fentanyl patch.**

**a. What dose of the fentanyl patch should be used?**  *(circle the correct answer)*

12 / 25 / 50 / 75 / 100 / 125 / 150 μg per hour / don’t know

**b. How would you make such a calculation for opioid rotation?**

❑ By heart

❑ I use the next resources as an aid: *(please circle the used aids)*

pain chart of the comprehensive cancer centers / pocket book palliative care / pharmacotherapeutic compass / own rules / other, namely: ………………….

❑ I consult the pharmacist

❑ I consult palliative care consultation team

❑ Another method, namely: ………………………………………………….

❑ I have no experience in this

VI. Tolerance

*By tolerance for a drug we mean that a patient needs a higher dose to reach the same pain relief while the pain stimulus remains the same. Tolerance has proven to be difficult to measure in practice, we are interested in your personal experience.*

*In daily practice, tolerance is difficult to objectify. We are interested in your personal experience.*

1. Please indicate how often (often, sometimes, seldom or never) the next statement are relevant in your practice.

|  | often | some times | seldom | never |
| --- | --- | --- | --- | --- |
| I have noticed that tolerance can develop in the usage of opioids | ❑ | ❑ | ❑ | ❑ |
| Tolerance hampers the usage of opioids in pain control | ❑ | ❑ | ❑ | ❑ |
| Patients' fear of addiction hampers the usage of opioids in practice | ❑ | ❑ | ❑ | ❑ |

VII. Sedation and shortening of life

**1. Please answer the next statements with ‘true’, ‘false’, or ‘don’t know’.**

*Circle the correct answer.*

**a.** Opioids titrated against pain, shorten life

true/ false/ don’t know

**b.** Opioids are the favoured drugs for palliative sedation

true/ false/ don’t know

**c.** Opioids are the appropriate drugs to perform euthanasia

true/ false/ don’t know

**2. Please indicate to what extend you agree with the next statements.**

Please answer these questions with a terminal patient in mind.

|  | fully agree | agree | neutral | disagree | fully disagree |
| --- | --- | --- | --- | --- | --- |
| Occasionally relatives of a patient or other persons concerned, put pressure on me to increase the opioids in the hope to hasten death | ❑ | ❑ | ❑ | ❑ | ❑ |
| When titrating the dosage of opioids upwards to control pain, I consider the possibility that this may hasten the death of the patient | ❑ | ❑ | ❑ | ❑ | ❑ |
| It occurs that I increase the dosage of opioids to a level above that of what is needed for pain and symptom control with the explicit aim to hasten the death of the patient. | ❑ | ❑ | ❑ | ❑ | ❑ |

VIII. Consultation

**1.** **In case you need consultation about pain management, how often do you consult the next persons?**

|  | often | some times | seldom | never |
| --- | --- | --- | --- | --- |
| Pharmacist | ❑ | ❑ | ❑ | ❑ |
| Regionally working palliative care consultation teams | ❑ | ❑ | ❑ | ❑ |
| Palliative team/consultant within hospital | ❑ | ❑ | ❑ | ❑ |
| Direct colleague own department/own practice | ❑ | ❑ | ❑ | ❑ |
| Anaesthesiologist | ❑ | ❑ | ❑ | ❑ |
| Oncologist | ❑ | ❑ | ❑ | ❑ |
| I search for information on the internet | ❑ | ❑ | ❑ | ❑ |

**2. For questions about palliative care a regionally working palliative care consultation can be contacted all over the Netherlands 24 hours a day.**

**Did you know about the consultation teams, and if so, have you ever once contacted them?**

❑ Yes, I have contacted the consultation team ever once.

❑ Yes, I know the consultation teams, but I have never contacted them because………………………..…………………………………………………

❑ No, I didn’t know the consultation teams

**3.** **Please indicate to what extend you agree with the next statements.**

Please answer these questions with a terminal patient in mind.

|  | fully agree | agree | neutral | disagree | fully disagree |
| --- | --- | --- | --- | --- | --- |
| Referral to a specialist feels like defeat | ❑ | ❑ | ❑ | ❑ | ❑ |
| Inadequate support from the pharmacist hampers my analgesics prescription | ❑ | ❑ | ❑ | ❑ | ❑ |

**4. Are unsolicited advice of the pharmacist concerning prescribed medicines appreciated?**

❑ Yes, because ………………………………………………………

❑ No, because …………………………………………………….

IX. General

**1.** **Please estimate the number of patients that died after a sickbed (not unexpectedly) in 2008 for whom you were (shared) involved in palliative care for this patient.**

none / 1-5 / 6-10 / 11-20 / 21-50 / over 50 *(patients per year*)

**2.** **What proportion of these patients used opioids at their tome of death?**

none / 1-20% / 21-40% / 41-60% / 61-80% / 81-99% / 100%

**3.** **Please remember the last patient whose death was expected and for whom you were responsible for providing palliative care**

**a.** **Did this patient use opioid(s)?**

*(multiple answers possible)*

❑ Yes, weak opioid(s) as tramadol, codein (as single drug or in combination)

❑ Yes, strong opioid(s), as fentanyl, morphine, oxycodon or methadon

❑ No

**b.** **Did this patient experience pain despite of pain management in the last 24 hours before death?**

❑ Yes, severe pain (6-10 on 10 points pain ladder)

❑ Yes, pain (3-5 on 10 points pain ladder)

❑ Hardly or no pain (0-2 on 10 points pain ladder)

❑ Not applicable (eg in the case of deep sedation)

X. Information and Education

**1. Did you, beside your regular education, receive any education in palliative care?**

❑ Yes, namely: *(multiple answers possible)*

❑ executive training palliative care

❑ postacademic education abroad

❑ symposia/conferences

❑ pharmacotherapeutic consultation between pharmacists and general practitioners (FTO)

❑ peer group

❑ other education, namely: …………………………………………………

❑ No

**2.** **I would like to receive education in the following topics:**

*(multiple answers possible)*

❑ Farmacology of opioids

❑ Opioid rotation

❑ Pain and symptom management in general

❑ Opioid side effects

❑ Palliative sedation

❑ Other subject(s), namely: ………………………………………………….

❑ No single subject related to opioids or pain management at the end of life

**3.** **Is there enough education available about opioids and pain management?**

❑ Yes, because …………………………………………………………………………

❑ No, because ………………………………………………………………………

**4.** **I prefer to be educated as follows:**

*(multiple answers possible)*

❑ Publications in Dutch journals

❑ Publication in international journals

❑ Other written sources (e.g. handbooks)

❑ Symposia/ conferences

❑ ‘Classic’ education by a teacher giving a lecture

❑ Knowledge transfer by peer groups

❑ Internet courses

❑ DVD’s or multimedia

❑ Other way of education, namely: ……………………………………………….

**5. How do you grade your own knowledge about opioids and pain management after filling out this questionnaire?**

……………………………*. (1 is lowest, 10 is highest )*

XI. Remarks

**Additional information and remarks**

…………………………………………………………………………………………..

…………………………………………………………………………………………..

…………………………………………………………………………………………..
